# Isolation of Bacteriophages with Lytic Activity from Biological Samples of Left Ventricular Assist Device Patients: An In Vitro Study

**DOI:** 10.3390/v18050526

**Published:** 2026-04-30

**Authors:** Balazs Sax, Adam Koppanyi, Katalin Kristof, Akos Kiraly, Gyula Prinz, Istvan Hartyanszky, Gergely Gyorgy Nagy, Istvan Nemet, Fanni Temesvari-Kis, Balazs Kiss, Bela Merkely

**Affiliations:** 1 Heart and Vascular Center, Semmelweis University, 1085 Budapest, Hungaryakos0009@yahoo.com (A.K.); hartyanszky.istvan@semmelweis.hu (I.H.);; 2 Institute of Laboratory Medicine, Semmelweis University, 1085 Budapest, Hungary; kristof.katalin@semmelweis.hu; 3 Centre for Cardiovascular Diseases and Internal Medicine, Borsod-Abauj-Zemplen County Central Hospital and University Teaching Hospital, 3526 Miskolc, Hungary; gergely.g.nagy@med.unideb.hu; 4 Fluart Innovative Vaccines Ltd., 2097 Pilisborosjeno, Hungary; istvan.nemet@fluart.hu (I.N.); fanni.temesvary-kis@fluart.hu (F.T.-K.); balazs.kiss@fluart.hu (B.K.)

**Keywords:** bacteriophages, biofilms, phage therapy, left ventricular assist device, driveline infection

## Abstract

Percutaneous cable infection of left ventricular assist device (LVAD) patients is a significant source of morbidity, often caused by biofilm-producing or multidrug-resistant bacteria. We hypothesized that bacteriophage viruses can be identified from biological samples of patients with active driveline infection. Six patients with local percutaneous lead infections were enrolled. Microbiological samples were collected from the infected wound and other skin regions. The isolated viral strains and phages from wastewater samples were then tested against the pathogen bacterial cultures in vitro. Biofilm disruption assay and genetic analysis of the strains were also performed. Bacteriophages with lytic activity could be identified from samples of two patients. One patient contained four strains showing strong efficacy against his own *Staphylococcus epidermidis*. Furthermore, this bacterium was susceptible to phages identified from another patient and strains from wastewater samples. Genomic analysis suggested lysogenic lifestyle of the phages. However, none of them have shown any microbiological signs of lysogeny. In conclusion, we have been able to prove in vitro lytic activity of bacteriophages originating from the same LVAD patient. We also found effective phages in biological samples of other patients and wastewater samples, suggesting that patients implanted in the same center may share bacteriophage flora.

## 1. Introduction

Long-term mechanical circulatory support is an increasingly available treatment option for patients with advanced heart failure. Despite vast development of the technology, the most cumbersome problem of left ventricular assist device (LVAD) patients remained driveline infection. This percutaneous cable connects the intrapericardial pump with the external controller and power source, typically exiting the subcutaneous tissue in the abdominal region. The exit point is the Achilles heel of technology: local bacterial infections may occur despite aseptic wound treatment.

Percutaneous cable exit site infections are often caused by multidrug-resistant and biofilm-producing bacteria, thus complete eradication of the pathogen is almost impossible in many clinical scenarios. Furthermore, they may ascend along the subcutaneous driveline tunnel and progress towards the central parts of the system. This can lead to bloodstream infections, eventually resulting in sepsis, requiring bailout solutions such as device exchange or urgent heart transplantation. Targeted antibiotic treatment and surgical wound care often only delay the progression of the disease, ultimately resulting in bloodstream or central component infection. Thus, novel approaches to treat driveline infection are sorely needed due to raising antibiotic resistance and persistent infection rates.

From an epidemiological perspective, the incidence of driveline infections range between 15% and 30% during the first two years after implantation [1,2]. Analyses of economic impact consistently show that infections related to LVADs, particularly those affecting the driveline, result in a considerable increase in healthcare resource utilization, including higher rates of readmission (up to 35% compared with 15% in non-infected patients), longer cumulative hospital stays, and increased utilization of diagnostic imaging, laboratory testing, and surgical interventions [3,4,5]. These factors directly contribute to increased costs: US-based estimates indicate that infection-related readmissions alone can add USD 30,000–60,000 per patient annually [6].

Cost-effectiveness models indicate that, while LVAD therapy remains justifiable in selected patient groups, driveline infections substantially erode its incremental cost-effectiveness ratio (ICER) by reducing quality-adjusted life years (QALYs) and increasing lifetime healthcare expenditures [6,7]. A body of research has demonstrated the efficacy of preventive strategies, including standardized driveline care protocols, antimicrobial dressings, and early-stage surgical revision, in reducing infection incidence and overall costs [5,8]. Furthermore, registry data demonstrate that infection-related adverse events are a significant contributor to mortality and morbidity post-LVAD, underlining the importance of preventive interventions not only for patient outcomes but also for the long-term economic sustainability of LVAD programs [9].

Bacteriophages are ubiquitous viruses that target and infect bacteria as their natural enemies. They are specific for certain bacterial strains and known to be harmless to eukaryotic cells, including human subjects. An important aspect of the life cycle of certain bacteriophages is the lysis of bacterial host cells, which is due to the rapid release of newly assembled viruses. As the lytic activity of bacteriophages is not limited or influenced by antibacterial resistance and phages can be effective against biofilm-producing pathogens, they are promising targets of antimicrobial research. There are a few case series available in the literature reporting the successful use of bacteriophage preparations in complicated driveline or LVAD infections [10]. The source of these intravenously or locally applied bacteriophage preparations are phage libraries, where effective strains can be selected from the available several virus strains. However, these libraries are not widely available. Moreover, it is technically very challenging, costly and time consuming to select clinically effective lytic phages by phenotypic screening alone.

As bacteriophages naturally occur in the surroundings of their target bacteria, there is a high chance of finding virus strains that might be effective weapons in the eradication of the culprit pathogen. In this proof-of-concept study, we hypothesized that bacteriophage viruses can be identified from biological samples of LVAD patients with active driveline infection that shows lytic activity against their own pathogen bacteria. Furthermore, we tested bacteriophages isolated from other patients of the center and also from commercial wastewater samples.

## 2. Materials and Methods

### 2.1. Patient Selection and Sampling

We included HeartMate3 LVAD patients with ongoing local (complicated and uncomplicated) percutaneous lead infections but without systemic infection. All patients had their drivelines externalized with a standard subcutaneous double tunnel technique. All had fixation of driveline with horizontal tube attachment device (Hollister #9781, Hollister Inc., Kirksville, MO, USA). Exit site wound care protocol included daily dressing change using octenidine dihydrochloride and phenoxyethanol spray (Octenisept, Schülke & Mayr GmbH, Norderstedt, Germany). Microbiological samples were collected using open-celled foam swabs and liquid amies media (Sigma-Σ-TRANSWAB^®^, Medical Wire & Equipment Ltd., Corsham, UK) from the following sites: infected wound, periumbilical region, armpit skin region, nasal cavity, and perianal region.

Infected wound samples were transferred to the Microbiology Laboratory of Semmelweis University, where they were cultured (under aerobic and anaerobic conditions), the pathogens were identified (MALDI-TOF MS, Biotyper^®^, Bruker, Billerica, MA, USA), and antibiotic susceptibility testing was performed according to good laboratory practice. The isolated bacteria and all other biological samples were then transferred to the bacteriophage laboratory where identification and purification of bacteriophage viruses took place.

### 2.2. Bacteriophage Isolation, Passaging and Propagation

Wet amies swabs of patient biological samples were immersed in 10 mL of Vegitone tryptic soy broth (TSB) (Merck KGaA, Darmstadt, Germany) and incubated overnight at 37 °C with agitation. Presence of effective phages were tested using spot test. Positive samples were then purified using double agar overlay technique. Pure phage population was obtained by three consecutive single plaque purifications. Purified phages were propagated double-agar overlay technique by using 10 mL of overlay agar in three replicates. After overnight incubation overlay agar layers were scraped and the plates were washed with phage buffer (100 mM TRIS, 50 mM NaCl, 10 mM MgCl_2_, 1 mM CaCl_2_, pH:7.4) overlay agars and washing buffers were merged and centrifuged (20 min, 7000× *g*, 4°C). Supernatants filtered using 0.22 μm syringe filters (Merck KGaA, Darmstadt, Germany) and titers were determined with spot assay method.

Wastewater samples were filtered through 0.22-μm syringe filters (Merck KGaA, Darmstadt, Germany) and diluted in equal amount of 2× Mueller Hinton broth and spiked with 10^7^ colony forming units (CFU)/mL of *Staphylococcus epidermidis* and incubated overnight at 37 °C with agitation. Supernatants were then filtered using 0.22-μm syringe filters (Merck KGaA, Darmstadt, Germany).

### 2.3. Host Range Characterization

The host range of selected phages was determined using the double-layer agar ‘spot test’ method. *Staphylococcus epidermidis* DSM 1798 (Leibniz Institute DSMZ-German Collection of Microorganisms and Cell Cultures GmbH) was used as the initial bacterial host for propagation of each phage to calculate and compare efficiencies of plating (E.O.P.). The concentration of each phage was normalized to ~10^9^ plaque-forming units (PFU)/mL. The infectivity of each phage, defined by the propensity to produce quantifiable plaques, was determined using a collection of 16 staphylococcal strains.

Briefly, overnight cultures of bacteria were mixed with soft TSA+CaCl_2_ to a concentration of ~5 × 10^7^ CFU/mL and then poured onto preset TSA plates. The top layer containing bacteria was allowed to dry, phages were serially diluted, and 10 μL of each dilution was plated onto the surface of the agar. Plates were incubated overnight at 37 °C. The limit for detection was 2.5 × 10^2^ PFU/mL.

### 2.4. Biofilm Disruption Assays

Biofilms were produced in 96-well microtiter plates and quantified essentially as described elsewhere [11]. Briefly, overnight cultures of bacteria were diluted 1:100 in TSB supplemented with 0.25% glucose (‘TSB+glucose’, 150 μL per well). Plates were incubated statically at 37 °C for 24 h. Following incubation, unattached cells were removed by inverting the plates, and then biofilms were washed by submerging the plates in sterile water. Approximately 1 × 10^8^ PFU of phages diluted in TSB were added (200 μL per well) and the plates were incubated for 4 h at 37 °C. SM-buffer was used for the untreated control. Following treatment, biofilms were washed three times then stained with crystal violet (CV; 0.06%) for 15 min at room temperature (RT). Excess CV was removed, and plates were again submerged three times in water. Biofilms were dried for 1 h at RT. Finally, acetic acid (30%) was added to each well and OD was measured at 550 nm (Thermo Scientific, Waltham, MA, USA). A total of five wells were used per condition, and each experiment was performed in biological triplicate. Comparisons between untreated and phage treated biofilms were determined using Kruskal–Wallis tests with multiple comparisons corrected for using Dunn’s multiple comparisons test. Data were considered significantly different when *p* ≤ 0.05.

### 2.5. Time–Kill Curves

Assays were carried out in 96-well microtiter plates and quantified via spectrophotometer (please provide exact modelThermo Scientific Multiskan Sky, Thermo Scientific, Waltham, MA, USA) at 600 nm using kinetic loop method. Briefly, overnight cultures of bacteria were diluted in TSB containing CaCl_2_ and MgCl_2_ to obtain 1 × 10^8^ CFU and loaded into 96-well plates alone or with different concentrations of patient phages or wastewater phages in 200 µL to obtain different multiplicity of infection (MOI: 1, 0.1, 0.01, and 0.001) in biological triplicates. Plates were incubated at 37 °C in a spectrophotometer (Thermo Scientific Multiskan Sky, Thermo Scientific) with pulsed shaking and optical density was measured in every two minutes for 20 h. OD600 was normalized with blank and the average of biological triplicates were visualized.

### 2.6. Phage Genome Sequencing and Analysis

Bacterial genomic DNA was extracted from ca. 50 mg cell pellet using the ZymoBIOMICS 96 MagBead DNA Kit (Zymo Research, Irvine, CA, USA) according to the manufacturer’s protocol. For lysis, bead beating was performed in Bashing bead lysis tubes in Omni Bead Ruptor Elite (OMNI International, Kennesaw, GA, USA) for 1 min on at 6 m/s then 5 min rest, repeat cycle 3 times for a total of 3 min of bead beating. Purified bacterial gDNA was eluted in 30 µL nuclease-free water.

To isolate bacteriophage genomic DNA, 0.5 mL of cell suspension was treated with 20 µL DNase I (Cat# E1010, Zymo Research) for 15 min at 37 °C followed by inactivation for 5 min at 75 °C. Then 10 µL of Proteinase K (Cat# ST-0106124, Revvity, Waltham, MA, USA) was added and cells were incubated for 30 min at 55 °C. 200 µL of this suspension was used for extraction without lysis by using ZymoBIOMICS 96 MagBead DNA Kit (Zymo Research).

DNA concentration was measured by Quant-iT 1x dsDNA BR Assay kit (Thermo Fisher Scientific, Waltham, MA, USA) with Infinite Pro 200 F Nano+ Fluorescent Plate Reader (Tecan, Männedorf, Switzerland). For shotgun DNA-Seq library construction, NEXTFLEX^®^ Rapid XP v2 DNA-Seq Kit with UDIs (Revvity, Waltham, MA, USA) was applied according to the manufacturer’s instructions. The fragment size distribution was determined by capillary electrophoresis on the LabChip GX Touch HT Nucleic Acid Analyzer (Revvity) with an X-Mark HT chip and the DNA NGS 3K Assay kit (Revvity). The library quantities were measured by Equalbit 1x dsDNA HS Assay Kit (Vazyme, Nanjing, PRC) with Infinite Pro 200 F Nano+ Fluorescent Plate Reader (Tecan).

PP01–02 phage genomes were sequenced using high-throughput Illumina sequencing on a NovaSeq 6000 [please include exact model, e.g., MiSeq/NextSeq 2000/NovaSeq 6000] (Illumina, San Diego, CA, USA), PP03–PP06 and WP01–WP04 phages were sequenced using Oxford Nanopore long-read technology on a GridION[include exact model here, e.g., MinION, GridION] device (Oxford Nanopore Technologies, Oxford, UK). Detailed methods for both sequencing platforms and bioinformatic analysis are described separately in Appendix A.

## 3. Results

### 3.1. Patient Demographics

Six LVAD patients with active driveline infection were included in this proof-of-concept study at a median 1985 (408-2963) days on LVAD support (Table 1).

All had none-to-mild CRP elevation, none of them had fever or positive hemoculture in their medical history related to LVAD infection. Two patients were taking systemic antibiotics at the time of sampling. Pathogen bacteria could be identified in five patients including *Staphylococcus epidermidis* and *Staphylococcus aureus*, *Dermabacter hominis*, *Corynebacterium striatum,* and *Serratia marcescens*.

### 3.2. Bacteriophage Identificaton

Bacteriophage strains with lytic activity could be identified in two of the six patients (Table 2). Biological samples of VM002 patient contained six lytic strains (PP01–06) showing efficacy against his own *Staphylococcus epidermidis* pathogen. Two further bacteriophage strains effective against the *S. epidermidis* of VM002 could be identified from samples of VM001 patient. The pathogens of three patients (*S. aureus*, *S. epidermidis*, *Serratia marcescens*) were susceptible to the lytic activity of multiple strains isolated from communal wastewater samples. Four of these phages effective against the *S. epidermidis* of VM002 were further studied in (WP01–04). In further analysis, we have performed host-range characterization, time–kill curves and detailed genetic analysis of the patient and wastewater derived bacteriophages against *S. epidermidis* of VM002.

### 3.3. Host Range Characterization

From the 16 tested *S. epidermidis* strains 9 showed response to at least some of the PP phages and 7 responded to at least any of the WP phages. *S. epidermidis* from VP002 patient responded to all the tested phages as shown in Table 3. Interestingly, the partially responding strains could be clustered in two groups by only responding to PP phages or only to WP phages.

### 3.4. Time-Kill Curves

**Figure 1:**
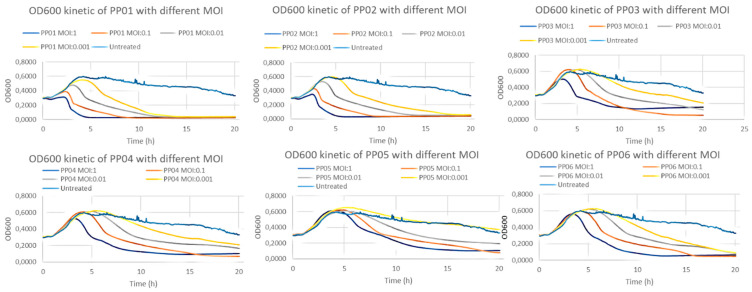
Time-kill curves of patient sample-derived bacteriophages (PP01–06).

All six patient sample-derived (PP) bacteriophages had been able to completely eliminate *S. epidermidis* strain of patient VM002 within 24 h in MOI:1 concentration. PP01 and PP02 had this effect even in low (MOI:001) concentration (Figure 1).

**Figure 2:**
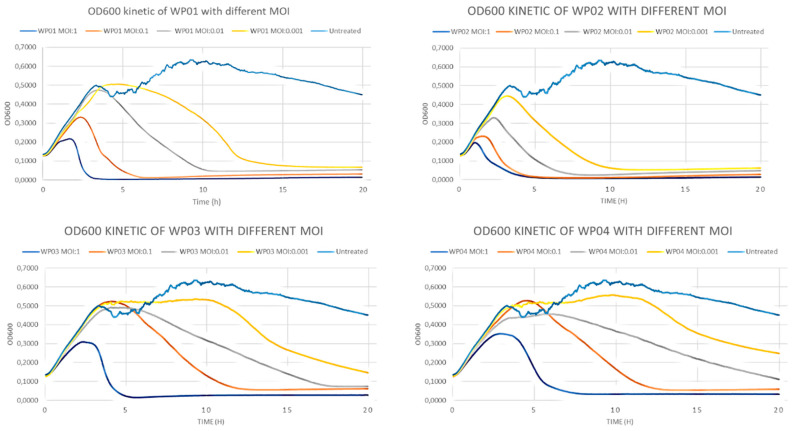
Time-kill curves of wastewater bacteriophages (WP01–04) on *S. epidermidis* strain of VM002 patient.

Similarly, all wastewater-derived (WP) bacteriophages were able to completely eliminate the *S. epidermidis* in MOI:1 and MOI:0.1 dilution. WP01 and WP02 had this effect even in low concentration (MOI:001) (Figure 2).

### 3.5. Effectivity Against Biofilm

**Figure 3:**
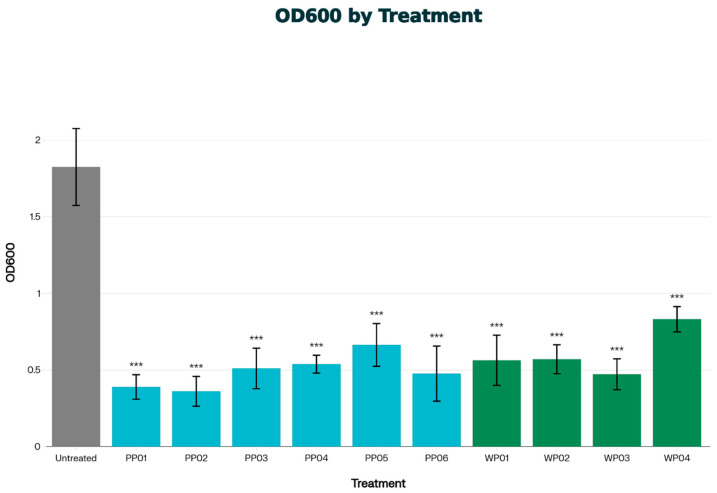
Biofilm disruption assay on *S. epidermidis* strain of VM002 patient. Lower optical density (OD) values indicate stronger effect on biofilms (*** *p* < 0.001 vs. untreated).

In biofilm disruption assays all tested bacteriophages with patient or wastewater origin (PP01–06 and WP01–04) showed significant biofilm disruption effect on *S. epidermidis* of VM002 patient as shown in Figure 3.

### 3.6. Taxonomic Assignment of Isolated Bacteriophages

Genome-based classification (TaxMyPhage, Pharokka, Phabox) placed all the isolates within the class of *Caudoviricetes* (phylum: *Uroviricota;* kingdom: *Heunggongvirae*; realm: *Duplodnaviria*), with *Staphylococcus epidermidis* predicted as the most probable host (CHERRYScore 1.0). Mash distance, average nucleotide identity (73–83%), and genomic clustering analyses confirmed that isolates are closely related to *Sextaecvirus* phages. However, they show enough differences to be considered unique members of this genus. Phylogenetic analysis and comparative genomics of the isolated bacteriophages can be found in Appendix A.

### 3.7. Comparative Genomics

**Figure 4:**
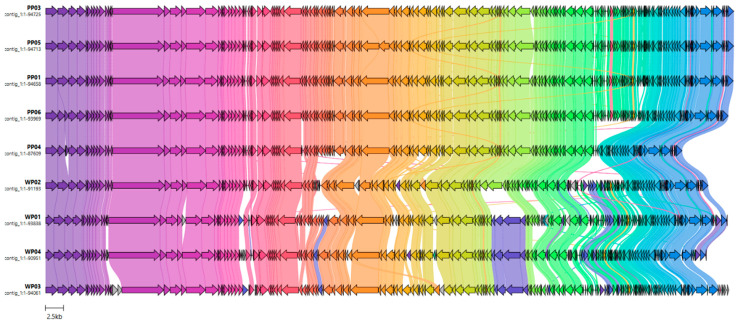
Comparative genomics of phages. Color-coded blocks represent identical regions and conserved gene clusters, while discontinuities and unique blocks indicate the presence of local rearrangements, insertions, or deletions.

A comparative genomic analysis of the isolates was performed and visualized by clinker, revealing extensive conservation of genome structure across all sequences (Figure 4). The core genome segment is maintained among all isolates. The occurrence of isolate-specific regions may reflect lineage-specific adaptive gene content. Genome completeness assessed by CheckV (v1.0.3, [12]) showed nearly complete assemblies for all isolates. PP04 and WP04 reached 95% and 98% completeness, respectively. All other genomes were classified as 100% complete.

### 3.8. Lifestyle of Phages

Functional annotation using PHASTER found an intact integrase gene in all isolates, indicating lysogeny. Similarly, PHAbox classified all the phage genomes as having a “temperate” lifestyle with high confidence. The PhaTYPScore was 1.0 for all phages, except for WP04, which had a PhaTYPScore of 0.59. The presence of integrase and the attL/attR attachment sites supports the possibility of prophage integration into the *S. epidermidis* host chromosome. Although the lytic module is intact and there are no antimicrobial resistance or virulence genes, these findings show that PP01–PP06 and WP01–WP04 are all temperate bacteriophages and they can maintain a lysogenic state in *S. epidermidis.*

## 4. Discussion

Percutaneous lead infection of LVAD patients is a common and cumbersome problem, as pathogens may form biofilm and tend to become multi-drug resistant. Bacteriophage therapy is a promising solution, as these viruses are not harmful to humans, their lytic activity is not affected by antibiotic resistance, and phages often show efficacy against biofilm [13]. There are a few case reports available where bacteriophage products were used in LVAD associated infections [14]. Most of these applications were applied in a more advanced stage of the disease, often as a bail-out solution when not only the percutaneous cable, but central components of the LVAD system were infected. Successful outcomes have been reported in combination with surgical debridement and antibiotic treatment against *Staphylococcus aureus* [15], *Proteus mirabilis* [16], and *Pseudomonas aeruginosa* [17]. However, in a case series where phage cocktails were applied intravenously against Pseudomonas infection, no successful outcome could be reported due to breakthrough bacteremia and serum neutralization [18]. The best practice for bacteriophage application (local vs. intravenous, which severity of mechanical circulatory support infections, autophages vs. lytic cocktails, etc.) in LVAD infection remains to be elaborated.

The bacteriophage preparations applied in the cited case reports were obtained from bacteriophage libraries in Georgia, Belgium or the United States, and commercially available virus cocktails were also used. In the present proof-of-concept study we have been able to identify, amplify and prove in vitro lytic activity of bacteriophages originating from biological samples of the infected patient. Furthermore, we were able to show that bacteriophages originating from other patients’ biological samples or from commercial wastewater may pose lytic activity against their pathogen.

These possibilities hold a promise to find lytic phage strains more effectively and—even more importantly—in a less time-consuming process compared to therapies where appropriate phages must be acquired from distant libraries. The interesting finding of effective phages in samples of other patients of the same center may open the possibility to set up center specific bacteriophage libraries. However, the in vivo activity and possibility of clinical application of the identified phages are yet to be determined.

Our results indicated that phages isolated from VM002 patient (PP01–PP04) showed similar patterns in terms of bacterial response as phages from different patient (PP05–PP06), but this pattern was different compared to phages from wastewater samples (WP01–WP04).

Time-–kill curve experiments showed that all studied phages were able to completely eliminate the *S. epidermidis* strain of patient VM002 up to 10^8^ PFU in a concentration of MOI:1. Some of the bacteriophages were able to do so even in very low concentration of MOI:0.001. In the examined period of 20 h there were no signs of regrowth. The results of biofilm disruption assay indicate that these phages could also be useful against biofilms formed on the surface of the percutaneous cable.

Based on life-cycle predictions and bioinformatic analysis all identified phages harbor the integrase gene as a key indicator of lysogen conversion. However, none of them have shown any signs of lysogenicity: we could not detect turbid plaque morphology, rims around clear plaques or halo formation during isolation, propagation and characterization of these phages. Besides these, strong lytic activity against many different strains and the fact that many of these phages were able to completely eliminate up to 10^8^ PFU of VM002 *S. epidermidis* within only 15 h indicates the dominance of lytic life cycle. Previous studies have also shown promising results using temperate phage in therapeutic applications [19]. Following in vitro amplification, extensive purification (filtration and removal of endotoxins and bacterial DNA) and safety characterization (genome sequencing and sterility testing), the autologous therapeutic application of these bacteriophages to same patient from whom these were harvested and isolated could be an acceptable option for compassionate use in rescue situations, where other treatment modalities failed. Moreover, targeted use of CRISPR-CAS9 system to remove integrase gene from tempered bacteriophages may also be a solution to prevent lysogenic life cycle [20]. Finally, these engineered bacteriophages in combination with synergistic antibiotic treatment could offer personalized treatment to the vulnerable patient population living with long-term mechanical circulatory support.

While the results of this in vitro study are promising, the study clearly has limitations. The safety and efficacy of clinical application is yet to be determined. The six patients included had different pathogens, but we were only able to find phages against *S. epidermidis* in patient derived samples. Furthermore, not all phages identified from patient or wastewater derived samples have shown proper lytic activity, and, therefore, only phages effective against the *S. epidermidis* strain of VM002 patient were studied further. Thus, not all bacteriophages found in the site of infection may pose in vivo activity on the pathogen bacteria.

## 5. Conclusions

In conclusion, our results suggest that it is feasible to identify effective bacteriophage strains with strong lytic activity from biological samples of patients with percutaneous lead infection. Furthermore, we were able to find viruses in biological samples of LVAD patients that are effective against pathogens of other patients, which suggest that patients implanted in the same center may share bacteriophage flora. Phages identified from wastewater samples may also serve as a promising solution where viruses from own biological samples cannot be identified. Our results provide the basis for developing novel antimicrobial strategies in LVAD patients.

## Figures and Tables

**Table 1:** Demographic data of patients and status of percutaneous lead infection.

Patient Number	Age (Years)	Sex	LVAD Support (Days)	Extent of Percutaneous Cable Infection	CRP (mg/L)	Antibiotic Treatment at Sampling
VM001	63	male	1665	exit site	10.1	cefixime p.o. 2 × 200 mg
VM002	60	male	1590	exit site	2.2	doxycycline p.o. 2 × 100 mg
VM003	39	female	408	exit site and tunnel turning point	3.2	none
VM004	71	male	2305	exit site	3.0	none
VM005	64	female	2963	widespread infection of the whole extrathoracic part	17.8	none
VM006	51	male	2901	exit site	3.4	none

**Table 2:** Results of bacteriophage identification from biological samples of the same or other patient (patient phage, PP) and from wastewater sample (wastewater phage, WP).

Patient Number	Pathogen Bacteria	Bacteriophages Identified from Biological Samples of the Same Patient	Bacteriophages Identified from Biological Samples of Other Study Patients	Bacteriophages Isolated from Wastewater Samples
VM001	none	-	-	
VM002	*Staphylococcus epidermidis*	6 strains (PP01–06)	2 strains from samples of VM001	4 strains (WP01–04)
VM003	*Staphylococcus aureus*	-	-	2 strains
VM004	*Dermabacter hominis*	-	-	
VM005	*Corynebacterium striatum*	-	-	
VM006	*Serratia marcescens*	-	-	3 strains

**Table 3:** Response of different *S. epidermidis* strains to the isolated patient (PP) and wastewater (WP) derived bacteriophages.

	PP01	PP02	PP03	PP04	PP05	PP06	WP01	WP02	WP03	WP04
*S. epidermidis* 1	+	+	+	-	+	-	-	-	-	-
*S. epidermidis* 2	-	-	-	+	-	+	-	-	-	-
*S. epidermidis* 3	-	-	-	-	-	-	+	-	-	+
*S. epidermidis* 4	+	+	+	+	+ +-	+	-	-	-	-
*S. epidermidis* 5	-	-	-	-	-	-	+	-	-	+
*S. epidermidis* 6	-	-	-	-	-	-	-	+	-	-
*S. epidermidis* 7	-	-	-	+	-	-	-	-	-	-
*S. epidermidis* 8	+	+	-	-	-	-	-	-	-	-
*S. epidermidis* 9	+	+	+	-	-	-	-	-	-	-
*S. epidermidis* 10	-	-	-	-	-	-	-	-	+	-
*S. epidermidis* 11	-	-	-	+	-	+	-	-	-	-
*S. epidermidis* 12	+	+	+	-	+	-	-	-	-	-
*S. epidermidis* 13	-	-	-	-	-	-	+	-	-	+
*S. epidermidis* 14	+	+	+	+	+	+	-	-	-	-
*S. epidermidis* 15	+	+	-	+	-	-	-	-	-	-
*S. epidermidis* 16	-	-	-	-	-	-	+	+	-	+
*S. epidermidis* VM002	+	+	+	+	+	+	+	+	+	+

**Figure A1:**
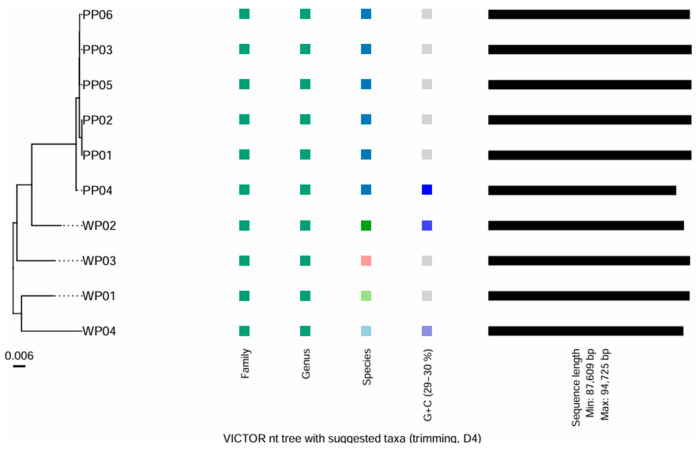
Phylogenetic analysis of the isolated phages.

**Figure A2:**
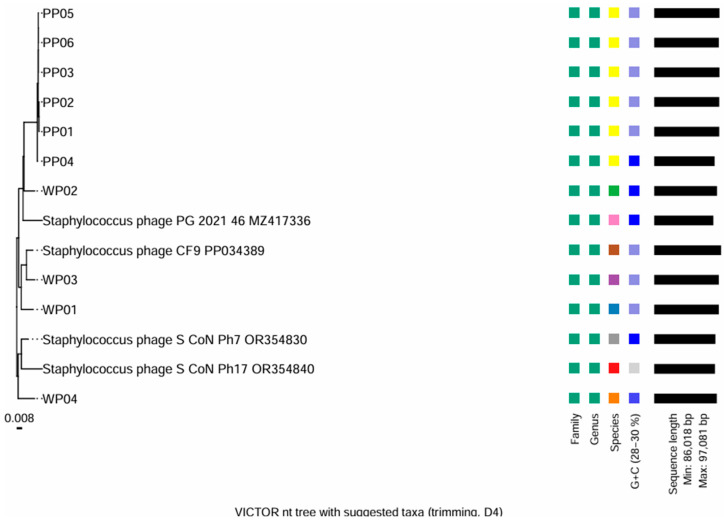
Phylogenetic analysis of the phage compared to most common *S. epidermidis* phages.

**Figure A3:**
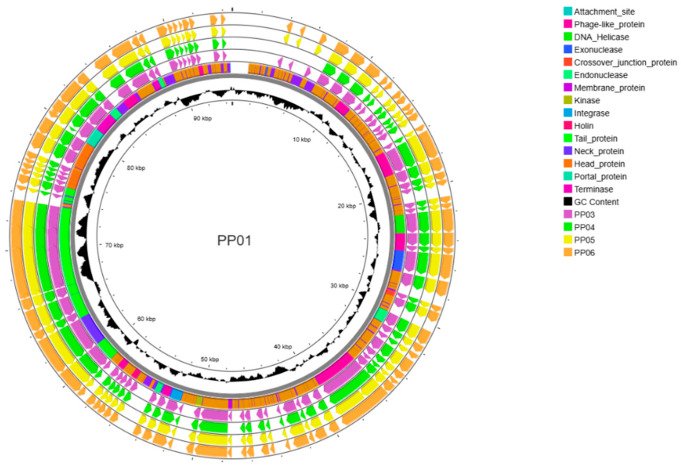
Genome organization of PP phages.

**Figure A4:**
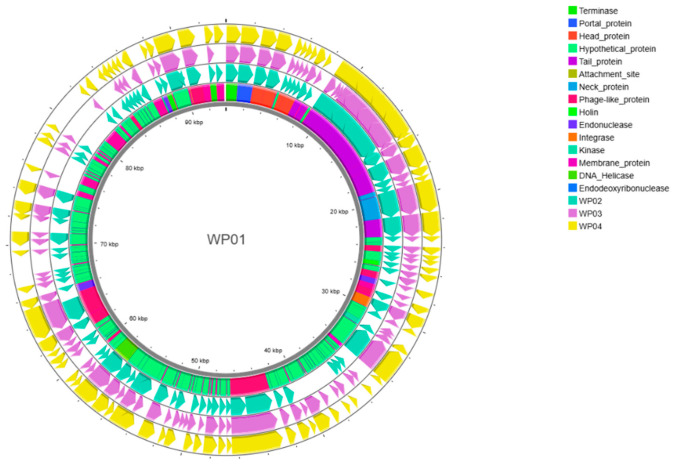
Genome organization of WP phages.

## Data Availability

The original contributions presented in this study are included in the article. Further inquiries can be directed to the corresponding author.

## Abbreviations

The following abbreviations are used in this manuscript:

CDSs	protein-coding sequences
CFUs	colony-forming units
CRP	C-reactive protein
CV	crystal violet
EOP	efficiencies of plating
ICER	incremental cost-effectiveness ratio
LVAD	left ventricular assist device
MOI	multiplicity of infection
OD	optical density
PFUs	plaque-forming units
PP	patient-derived phage
QALY	quality-adjusted life years
RT	room temperature
TSB	tryptic soy broth
WP	wastewater-derived phage

## Appendix A

### Appendix A.1. Phage Genome Sequencing and Analysis

#### Appendix A.1.1. Genome Characterization of PP01

High-throughput Illumina sequencing and quality filtering produced approximately 36 million paired-end reads per direction. The average Phred quality score exceeded Q30 for all positions. De novo assembly with SPAdes resulted in a contiguous sequence of 94,658 bp, representing the complete phage genome, with a GC content of 29% and an estimated coverage depth of 700×. CheckV classified the assembly as high quality, with 100% completeness and no detectable host contamination. No antimicrobial resistance or virulence factor genes were found (CARD, ResFinder, VFDB).

#### Appendix A.1.2. Genome Characterization of PP02

High-throughput Illumina sequencing and quality filtering produced about 356,527 paired-end reads per direction (average length 122 bp), with an average Phred quality score over Q30 for all positions. The initial de novo assembly with SPAdes produced two contigs of medium and low quality according to CheckV. This led to a reference-guided assembly using the PP01 genome, resulting in a contiguous sequence of 94,665 bp and a GC content of 29%. CheckV classified the final reference-based assembly as high quality, with complete genome recovery and no detectable host contamination. Variant calling found no differences, confirming 100% sequence identity between the PP02 and PP01 genomes.

#### Appendix A.1.3. Genome Characterization of PP03–PP06 and WP01–WP04

Oxford Nanopore long-read sequencing of phages PP03-WP04 produced high-quality long-read datasets, suitable for de novo genome assembly. Across the eight samples, mean read qualities remained consistently around Q20 (range: 19.9 to 20.7), with total bases ranging from 22.2 Mb to 85.1 Mb and the number of reads from 12,763 to 100,517. Q30+ reads made up 28 to 42% of the datasets, and the longest reads often exceeded 40 to 80 kb, with top basecall qualities near Q48. These long, high-quality reads provided enough depth and contiguity for complete or nearly complete de novo assembly of the PP03-WP04 genomes using long-read assemblers designed for Oxford Nanopore data.

#### Appendix A.1.4. Quality Control and Assembly for PP01 and PP02 Phages

Raw sequencing reads in fastq format were first subjected to quality assessment using FastQC (v0.12.1). Low-quality bases and adapter sequences were removed and the overall quality was improved by trimming and filtering raw reads using fastp (v0.23.2, [21]).

Prior to assembly, GenomeScope 2.0 [22] was used for k-mer frequency analysis to estimate genome size, sequencing coverage, potential error rates, genetic diversity, and repetitive content from filtered reads.

The complete PP01 phage genome was assembled de novo using SPAdes (v4.0.0, [22]) with “--careful” mode to minimize mismatches and short indels, producing contiguous sequences suitable for downstream genome annotation.

The PP02 phage genome was assembled using a reference-guided approach since the initial de novo assembly produced two contigs with medium and low quality, as indicated by CheckV (v1.0.3, [12]). The PP01 genome was used as the reference genome. Trimmed and quality-filtered paired-end reads were aligned to the reference sequence using BWA MEM v0.7.19 [23]). Resulting SAM files were converted, sorted, and indexed into BAM format with Samtools v1.22 [24,25]. Variants relative to the reference were called using bcftools v1.14 mpileup and call, with compressed and indexed VCF files generated for each sample [25]. Consensus sequence was generated from the variant calls using bcftools consensus. This reference-based assembly approach improved sequence quality compared to the original de novo assembly.

Assembly completeness and potential host contamination were evaluated using CheckV (v1.0.3, [12]), while overall assembly quality assessment for PP01 was generated using QUAST (v5.0.2, [26]).

#### Appendix A.1.5. Quality Control and Assembly for PP03–PP06 and WP01–WP04 Phages

Nanopore-specific quality assessment was performed using NanoPlot [27], which provided an overview of read length and quality profiles, helping to determine the filtering parameters for the next steps. Adapters were removed using Porechop (v0.2.4, [28]), followed by read filtering with Filtlong (v0.3.1., [29]). This filtering step retained reads with a minimum length of 500 bp while prioritizing high-quality reads aiming for a maximum of 40 Mb per sample. The filtered long reads were assembled de novo using Flye (v2.9.6, [30]), applying parameters optimized for Nanopore sequencing data to generate contiguous phage genome assemblies. To correct residual Nanopore-specific errors, each assembly was polished using three rounds of Racon (v1.5, [31]). In each round, filtered reads were mapped back to the corresponding Flye assembly using minimap2 (v2.30, [32]), followed by consensus correction with Racon. The final assemblies were further polished using Medaka (v2.1.0, Oxford Nanopore Technologies, 2020).

#### Appendix A.1.6. Annotation and Genome Organization

The phage genomes were annotated using a combination of Pharokka (v1.7.5, [33]) and PHASTER [34]. Genomes were screened for antibiotic resistance genes using the Comprehensive Antibiotic Resistance Database (CARD; [35]) and ResFinder 1.2. [36], and for virulence genes using the VFDB database [37]. Circular representations of phage genomes were created using Proksee [38].

#### Appendix A.1.7. Bacteriophage Classification and Lifestyle Prediction

The taxonomic classification of our phages was performed using TaxMyPhage (v0.3.6, [39]), PhaBOX (v2.1.5, [40]) and Pharokka (v1.7.5, [33]). For the prediction of the phage lifestyle (lytic versus temperate) PhaBOX (v2.1.5, [40]) was used, which integrates genomic feature analysis with machine learning classification.

Phylogenetic analysis was carried out by the VICTOR web service (https://victor.dsmz.de; accessed on 23 March 2026), a method for the genome-based phylogeny and classification of prokaryotic viruses [41]. All pairwise comparisons of the nucleotide sequences were conducted using the Genome-BLAST Distance Phylogeny (GBDP) method under settings recommended for prokaryotic viruses [41,42].

The resulting intergenomic distances were used to infer a balanced minimum evolution tree with branch support via FASTME including SPR postprocessing for each of the formulas D0, D4 and D6, respectively [43]. Branch support was inferred from 100 pseudo-bootstrap replicates each. Trees were rooted at the midpoint and visualized with ggtree [44,45].

Taxon boundaries at the species, genus and family level were estimated with the OPTSIL program [46], the recommended clustering thresholds and an F value (fraction of links required for cluster fusion) of 0.5 [41,47].

#### Appendix A.1.8. Comparative Genomics

Comparative genomics of P03–P06 and WP01–WP03 phages were carried out at the protein level using Clinker v0.0.27 [48]. Predicted protein sequences from the annotated genomes were clustered by sequence similarity, and Clinker was used to generate a plot highlighting conserved gene blocks and rearrangements among the studied phages.

### Appendix A.2. Results—Bioinformatic Analysis

#### Appendix A.2.1. Phylogenetic Analysis

In the D4-trimmed VICTOR tree PP01–PP06 and WP01–WP04 form two main clusters within the Sextaecvirus group, mirroring the separation between PP- and WP-type phages. PP01, PP02, PP05, and PP03 occupy a closely related lineage that branches together with PP06 and PP04, consistent with their highly similar genome organization and GC content. In contrast, WP01, WP02, WP03, and WP04 group in a separate, but still Sextaecvirus-associated clade, indicating that they represent a distinct, though related, genomic subgroup within the same genus. The short internal branches suggest strong genomic conservation and recent common ancestry among these isolates (Figure A1). Both PP and WP lineages are clearly related to the closest reference Sextaecvirus genomes included in the analysis, supporting their classification within the same genus but in partially differentiated sublineages (Figure A2).

#### Appendix A.2.2. Genome Organization and Functional Modules

Annotation with Pharokka (v1.7.5, [33]) predicted 150–181 protein-coding sequences (CDSs) in the isolates and one tRNA gene, yielding a coding density of 93%. Genome organization is shown for patient-derived phages in Figure A3 and wastewater-derived phages in Figure A4. Functional grouping of genes for the ten analyzed bacteriophages are detailed in Table A1.

**Table A1:** Functional grouping of genes.

Module	Number of Genes/Features								
	PP01	PP03	PP04	PP05	PP06	WP01	WP02	WP03	WP04
CDS	181	180	150	180	176	174	172	175	168
Connector	5	5	5	5	5	6	5	5	5
DNA, RNA, and nucleotide metabolism	20	20	20	21	20	20	22	23	20
Head and packaging	5	5	5	5	6	6	5	5	6
Integration and excision	0	0	0	0	0	0	0	0	0
Lysis module	2	2	2	2	2	3	2	3	3
Moron, auxiliary metabolic gene, and host takeover	6	6	5	5	6	6	5	5	5
Other	7	7	6	7	7	6	7	6	6
Tail morphogenesis	8	8	8	8	8	8	8	9	8
Transcription regulation	2	2	2	2	2	2	3	2	2
Unknown function	126	125	97	125	120	117	115	117	113
tRNAs	1	1	1	1	1	1	1	1	1
CRISPRs	0	0	0	0	0	0	0	0	0
tmRNAs	0	0	0	0	0	0	0	0	0
Virulence Factors (VFDB)	0	0	0	0	0	0	0	0	0
AMR Genes (CARD)	0	0	0	0	0	0	0	0	0
